# Should ‘typical’, first-generation antipsychotics no longer be generally used in the treatment of schizophrenia?

**DOI:** 10.1007/s00406-021-01335-y

**Published:** 2021-09-29

**Authors:** Stefan Leucht, Maximilian Huhn, John M. Davis

**Affiliations:** 1 Section for Evidence Based Medicine in Psychiatry and Psychotherapy, Department of Psychiatry and Psychotherapy, Technical University of Munich, School of Medicine, Ismaninger Straße. 22, 81675 Munich, Germany; 2 Department of Psychiatry, Psychosomatic Medicine and Psychotherapy, Social Foundation Bamberg, Teaching Hospital of the University of Erlangen, Erlangen, Germany; 3 Psychiatric Institute, University of Illinois at Chicago, Chicago, IL USA; 4 Department of Psychiatry, John Hopkins University, Baltimore, MD USA

Since the reintroduction of clozapine in many countries after the landmark trial by Kane et al. in 1988 [1], there are, to date, 16 second-generation, ‘atypical’ antipsychotics in the United States and/or in Europe. The World Health Organization currently lists an additional 52 antipsychotic drugs which are usually classified as first-generation or ‘typical’ [2]. In the 1990s and 2000s, there was a heated debate as to whether the much more costly, second-generation antipsychotics should be preferred to the cheaper first-generation drugs. The essential conclusion of this debate was that overall SGAs produce fewer movement disorders but more weight gain than FGAs, and that some SGAs were somewhat more efficacious than FGAs. However, there are too many exceptions from this rule (e.g. the FGA thioridazine produces very few movement disorders while the SGA risperidone produces a considerable amount, ziprasidone does not produce significant weight gain while chlorpromazine does) for this classification to be valid. Therefore, the misleading and wrong ‘typical’/FGA versus ‘atypical’/SGA distinction has been abandoned and replaced by the Neuroscience-Based Nomenclature [3] which classifies drugs according to their primary mechanism of action.

A decade later, many of the second-generation antipsychotics have lost their patent protection and are available as cheaper generics. One may thus ask whether we should not abandon FGAs altogether? The answer is, it depends on the drug.

On the one hand, the cost debate is not over everywhere. For example, one dose of olanzapine 10 mg i.m. costs 30€ in contrast to only 3€ for haloperidol 5 mg i.m. in Germany, and in developing countries much smaller differences may be important. Thus, FGAs are necessary for medico-economic reasons in many countries and haloperidol, chlorpromazine and fluphenazine together with risperidone and clozapine are on the WHO’s list of essential drugs [4]. This list presents drugs which should be available as a minimum in every country.

**Fig. 1:**
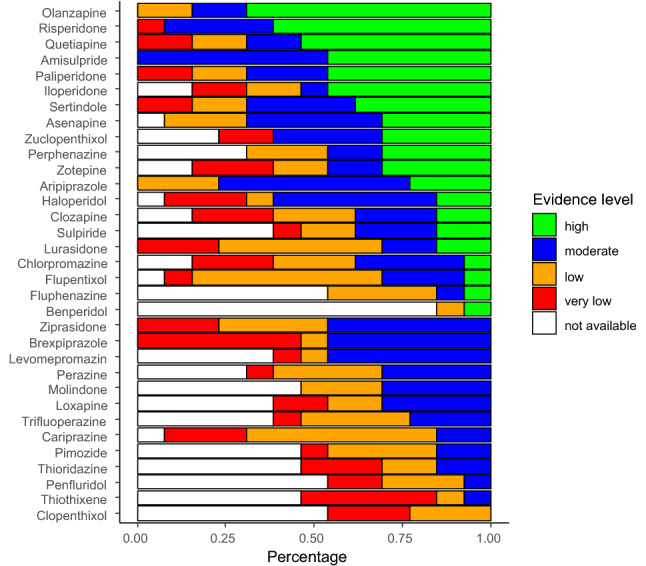
Confidence in evidence for antipsychotic drugs compared to placebo in terms of 13 outcome measures. White bars indicate the percentage of outcomes for which not a single RCT was available (figure reproduced with permission from Huhn et al. 2019 [5]). In this figure published in Huhn et al. 2019 [5] for every antipsychotic drug the percentage of outcomes with the following evidence levels was presented: high = blue, moderate = green, low = orange, very low = red according to CINeMA (Confidence in Network Meta-analysis). If outcomes were not reported in at least a single trial, we added them as “not available” in white. Benperidol, a frequently used drug in Germany, was added. The following 13 outcomes were used: overall symptoms, positive symptoms, negative symptoms, depression, all-cause discontinuation, quality of life, functioning, weight gain, use of anti-Parkinson medication, akathisia, sedation, QTc prolongation and prolactin increase

On the other hand, it needs to be emphasised that the older drugs have not been nearly as well studied as the newer drugs. When we examined multiple first-generation antipsychotics in our recent network meta-analyses on the acute treatment and the maintenance treatment of schizophrenia [5, 6], it turned out that for almost all FGAs, the number of trials and patients randomised were so small that even if they were combined in one trial they would nowadays not fulfill the criteria of a power calculation (see also Samara et al. [7] which compared chlorpromazine with any other FGA and found that almost all comparisons were underpowered). In the acute-phase network meta-analysis, the confidence in the evidence was very low to low for most first-generation antipsychotics. Indeed, for most FGAs, many of the 13 outcomes chosen for our meta-analysis were not analysed in a single trial. This finding is illustrated by the white bars in Fig. 1 which show the percentage of the 13 outcomes analysed in Huhn et al. [5] for which not a single trial was available.

Therefore, although some of these drugs have been available for more than 50 years, we know much less about them than about SGAs, and most of them would nowadays not be licensed by current standards. This lack of evidence can have consequences for patients. Thioridazine was used a lot in elderly patients until it turned out in studies on second-generation antipsychotics in the 1990s that it is the antipsychotic associated with most QTc prolongation. The most notable exception is haloperidol which was the gold-standard comparator in trials on second-generation antipsychotics and is, therefore, among the best examined antipsychotics. Thus, haloperidol is and will remain a standard antipsychotic. Another example is perphenazine, a FGA which was used more frequently than haloperidol in some Northern European countries in the “pre-atypical’’ era. Perphenazine was the comparator FGA in the large, industry-independent CATIE study in which it turned out to be as good if not better than several second-generation antipsychotics [8, 9]. Thus, at least one large trial meeting modern standards in which all relevant outcomes were examined is available. Moreover, only a few SGAs are available as short-acting or long-acting injectable antipsychotics or as liquids. The FGAs which can fill this gap should also not be abandoned. Pharmaceutical companies tend to give up old drugs when they cannot make much profit with them anymore. A recent painful case was the withdrawal of intravenous haloperidol due to QTc prolongation. As it had been a standard treatment for decades, it left a gap with few intravenous antipsychotic options left. Another example is perphenazine of which the long-acting formulation recently disappeared from German and other pharmacies.

We conclude that with few exceptions, SGAs should be preferred to most FGAs unless medico-economic reasons preclude the use even of SGA generics. Having said this, some of the older drugs have interesting properties such as unique receptor binding profiles. These FGAs might thus offer real clinical advantages and be cost effective if they were well studied. In our next editorial, we will, therefore, discuss which FGAs deserve to be studied further with the aim of ‘resuscitating’ them for practice.
